# Diurnal and Seasonal Variations in the Net Ecosystem CO_2_ Exchange of a Pasture in the Three-River Source Region of the Qinghai−Tibetan Plateau

**DOI:** 10.1371/journal.pone.0170963

**Published:** 2017-01-27

**Authors:** Bin Wang, Haiyan Jin, Qi Li, Dongdong Chen, Liang Zhao, Yanhong Tang, Tomomichi Kato, Song Gu

**Affiliations:** 1 Key Laboratory of Marine Ecosystem and Biogeochemistry, Second Institute of Oceanography, State Oceanic Administration, Hangzhou, China; 2 College of Life Science and Agriculture and Forestry, Qiqihar University, Qiqihar, China; 3 Northwest Plateau Institute of Biology, The Chinese Academy of Science, Xining, China; 4 National Institute for Environmental Studies, Tsukuba, Ibaraki, Japan; 5 Doctoral Program in Biological Sciences, University of Tsukuba, Tsukuba, Ibaraki, Japan; 6 College of Life Science, Nankai University, Tianjin, China; Beijing Normal University, CHINA

## Abstract

Carbon dioxide (CO_2_) exchange between the atmosphere and grassland ecosystems is very important for the global carbon balance. To assess the CO_2_ flux and its relationship to environmental factors, the eddy covariance method was used to evaluate the diurnal cycle and seasonal pattern of the net ecosystem CO_2_ exchange (NEE) of a cultivated pasture in the Three-River Source Region (TRSR) on the Qinghai−Tibetan Plateau from January 1 to December 31, 2008. The diurnal variations in the NEE and ecosystem respiration (R_e_) during the growing season exhibited single-peak patterns, the maximum and minimum CO_2_ uptake observed during the noon hours and night; and the maximum and minimum R_e_ took place in the afternoon and early morning, respectively. The minimum hourly NEE rate and the maximum hourly R_e_ rate were −7.89 and 5.03 μmol CO_2_ m^−2^ s^−1^, respectively. The NEE and R_e_ showed clear seasonal variations, with lower values in winter and higher values in the peak growth period. The highest daily values for C uptake and R_e_ were observed on August 12 (−2.91 g C m^−2^ d^−1^) and July 28 (5.04 g C m^−2^ day^−1^), respectively. The annual total NEE and R_e_ were −140.01 and 403.57 g C m^−2^ year^−1^, respectively. The apparent quantum yield (α) was −0.0275 μmol μmol^−1^ for the entire growing period, and the α values for the pasture’s light response curve varied with the leaf area index (LAI), air temperature (T_a_), soil water content (SWC) and vapor pressure deficit (VPD). Piecewise regression results indicated that the optimum T_a_ and VPD for the daytime NEE were 14.1°C and 0.65 kPa, respectively. The daytime NEE decreased with increasing SWC, and the temperature sensitivity of respiration (Q_10_) was 3.0 during the growing season, which was controlled by the SWC conditions. Path analysis suggested that the soil temperature at a depth of 5 cm (T_soil_) was the most important environmental factor affecting daily variations in NEE during the growing season, and the photosynthetic photon flux density (PPFD) was the major limiting factor for this cultivated pasture.

## Introduction

Grassland ecosystems occupy approximately one-third of the total global land area and form an important component of the earth’s carbon circulation [1]. During the past few decades, ecologists have studied the effects of environmental factors (such as radiation, temperature, water and soil nutrition), biological factors and management measures on the carbon exchange between the land surface and the atmosphere of the grassland ecosystem by using eddy covariance [2, 3], and these ecologists have noted the significance of human activity on the carbon exchange process [4, 5]. The grassland of China occupies approximately 40% of the nation's total land area and plays an extremely important role in the regional circulation of carbon [6]. However, because the study of China’s grassland carbon flux began late, these studies have mainly focused on the low-lying regions of China [7].

The Qinghai−Tibetan Plateau has drawn considerable attention as the “initiation zone” and the “sensitivity zone” for China’s weather changes [8, 9]. Although there have been reports on the process of carbon exchange between the land surface and the atmosphere and on the carbon exchange mechanisms of the primary natural vegetation types (e.g., alpine meadows and alpine shrubs) over the last several years [10, 11], there have only been a few reports on the carbon exchange process, the source/sink function of planted vegetation (e.g., cultivated grassland) and the mechanisms controlling the exchange among environmental and biological factors.

The Three-River Source Region (TRSR, i.e., the source of the Yangtze, Yellow and Mekong Rivers and well known as the “water tower of Asia”) is located in the hinterland of the Qinghai−Tibetan Plateau. In recent years, the grassland in this region has severely degraded. Statistics indicate that the area that is experiencing moderate and severe degradation has already reached 5.7×10^6^ hm^2^, occupying 55.40% of the total usable grassland area in this region [12]. This degradation can reduce vegetation biomass [13], soil microorganism activity [14] and soil carbon and nitrogen pools [13, 15] and can increase carbon dioxide (CO_2_) emissions [16]. It is estimated that during the last 30 years, approximately 1.01 Pg of soil carbon was emitted from the grasslands of the plateau due to changes in land use and grassland degradation [17]. Thus, grassland degradation on the Qinghai−Tibetan Plateau may have an important impact on the carbon balance at both the regional and global scales. To restore grassland, China’s largest demonstration area for “returning grazing land to grassland” was established in the TRSR. By 2005, the cultivated pasture area in the TRSR had already reached 160,000 km^2^ [18]. An increase in cultivated pasture may slow degradation and help restore the degraded rangelands [13, 19]. After moderate “disturbance”, restoration and rehabilitation, the degraded grassland ecosystems can alter the aboveground community and the soil properties and functions [15, 20]. Dong et al. (2012) [21] showed that the establishment of cultivated grassland on the degraded black soil grasslands in alpine regions of the Qinghai−Tibetan Plateau accelerated the vegetative succession and renewed the soil nutrient cycle, leading to a marked increase in carbon storage. However, it is not clear whether cultivated pasture acts as a CO_2_ sink or source.

We sought to identify the influence of the establishment of cultivated grassland on the ecosystem’s carbon budget. We wanted to understand the effects of environmental and biological factors on the carbon budget of the cultivated grassland, but there are few reports on these issues. Therefore, in the present study, we used an eddy covariance system to continuously collect observational data from January 1 to December 31, 2008, and performed a quantitative analysis of the CO_2_ flux variations and controlling factors in the TRSR pasture to achieve the following objectives: (1) the quantification of the magnitude of the diurnal and seasonal changes in the net ecosystem CO_2_ exchange (NEE) and ecosystem respiration (R_e_), (2) the examination of the dependence of carbon fluxes on abiotic and biotic factors and (3) the calculation of the carbon budget of the cultivated pasture during 2008.

## Materials and Methods

### Study site

The study area is situated in the Geduo pastoral pasture, 25 km southeast of the town of Dawu in Guoluo Prefecture in Qinghai Province, which is located in the TRSR. The geographical coordinates of the area are 100°26′–100°41′E and 34°17′–34°25′N, and the area lies at an elevation of 3980 m above sea level. The area experiences the continental weather typical of the plateau: the annual average sunshine duration is 2576 h, the radiation is strong, there is no absolute frost, and the annual average temperature is −0.5°C. The average temperature in January is −12.7°C, the average temperature in July is 9.8°C, and the annual precipitation (PPT) is approximately 500 mm, with 85% of the PPT concentrated between May and September. The soils are mainly an alpine meadow type and an alpine shrub type. The artificial pasture was established in May 2002, and its total area was 2000 hm^2^. The pasture was sown only with *Elymus nutans*, and the pasture vegetation height was 40 to 60 cm. During winter, the meadow was subjected to moderate-intensity grazing.

### Ethics statement

The study area was owned and/or managed by the Northwest Plateau Institute of Biology of Chinese Academy of Sciences, who gave permission to perform the field research. In the study area, no specific permits were required for collecting samples, and the field studies did not involve endangered or protected species.

### Eddy flux and micrometeorological measurements

An eddy covariance flux tower (3.0 m high) was installed at the center of the observation field. The fluxes of CO_2_ and H_2_O were measured using the eddy covariance method. The uniform fetch was more than 300 m from the tower in all directions. A three-dimensional ultrasonic anemometer, manufactured by Campbell Scientific, Inc. (CSI) (CSAT−3, Logan, UT, USA), was used to measure turbulence. The CO_2_ and H_2_O densities and the temperature fluctuations were measured using an open-path CO_2_/H_2_O infrared gas analyzer (CS7500, CSI) and an anemometer−thermometer at 10 Hz, respectively. The average value was output once every 15 min, and the data were saved in a data collection device (CR5000, CSI). The CO_2_/H_2_O analyzer system was calibrated each year.

Simultaneously with measuring the CO_2_ flux, we also measured other routine weather factors. The system for obtaining the routine measurements was installed on the same flux tower as the eddy measurement system. Among these measurements, the net radiation was measured using a net radiometer (CNR−1, Kipp and Zonen, Delft, South Holland, The Netherlands), and the photosynthetic photon flux density (PPFD) was measured using a quantum sensor (LI−190SB, Li−Cor, Lincoln, NE, USA). Both measurements were recorded at a height of 150 cm. The soil temperature was measured using copper−constantan thermocouples (105−T, CSI) at depths of 5, 10 and 30 cm underground. The air temperature (T_a_) and humidity were measured with a humidity and temperature probe (HUMP45C, CSI) at heights of 110 and 300 cm above the ground. The wind speed and direction were also measured at heights of 110 and 300 cm above the ground using cup anemometers (034A−L and 014A, R. M. Young Co., Traverse, MI, USA). The soil heat flux was measured at a soil depth of 2 cm with heat flux plates (HFT−3, CSI). In total, there were three heat flux plates in the test field, and the average of the soil heat flux values recorded by the three plates was used. The soil moisture was measured using time-domain reflectometers (CS615, CSI) at depths of 5, 20 and 30 cm underground. The soil surface temperature was measured with thermometers (107, CSI) at three points in an area of 1 m^2^. The PPT volume was determined using a tipping bucket (TE525MM, CSI) mounted 70 cm above the ground. The output data consisted of average values calculated every 15 min. These data were stored in the data collector (CR5000, CSI).

### Data processing and energy balance closure

The data were obtained from January 1 to December 31, 2008. All the micrometeorological and flux data were subjected to data quality control. The raw flux data were preprocessed before analysis, which primarily included outlier exclusion (±3δ), dimensional coordinate rotation and the application of the Webb−Pearman−Leuning correction [22], among others.

The surface energy budget of the sample field was examined by performing an ordinary linear regression (OLR) between the sum of eddy fluxes (LE+H) and the available energy (R_n_−G) during all of 2008: LE+H = 0.69×(R_n_−G)+22.06 (R^2^ = 0.84), where LE and H are the latent and sensible heat fluxes, respectively, R_n_ is the net radiation, G is the soil heat flux, and all the flux values are daily averages (MJ m^−2^). Therefore, the energy closure ratio was 69% for the sample field, and this energy closure slope is within the published energy closure range (0.55 to 0.90) [23]. Notably, the area contributing to the flux was large, flat and wide open, but the closure slope was relatively small. This difference might have been caused by the relatively low temperatures and wind speeds, and further study is needed to determine whether these weather conditions explain the discrepancy.

Because the flux observation and measurement are affected by the weather conditions at the site, the data can be processed by eliminating values collected during PPT, morning dew periods, and nights during the growing season when the carbon flux volume is negative (ecosystem carbon absorption). The data from the night times when the friction wind velocity (U*)≤0.2 m s^−1^ can be treated as invalid because the turbulence intensity at those times was not strong enough for the device to properly record CO_2_ flux data [24]. After processing, 67% of the total flux data collected were usable.

The data gaps that were less than 3 h in duration were filled through linear interpolation between the preceding and subsequent data. The missing data in gaps that exceeded 3 h could usually be interpolated based on the nonlinear empirical relation between the established carbon flux value and the environmental factors [25].

The nighttime data gaps were filled using the soil temperature at a depth of 5 cm (T_soil_) according to Formula (1), and daytime estimates of R_e_ could be obtained according to the nighttime R_e_−temperature relationship as follows [26]:

$$R_{e}=a\text{exp}(bT_{soil}),$$ (1)

where R_e_ is the nighttime R_e_ rate (μmol CO_2_ m^−2^ s^−1^), T_soil_ is the soil temperature at a depth of 5 cm, and a and b are fitted coefficients in Formula (1). The temperature sensitivity of respiration (Q_10_) for the ecosystem was derived from Formula (2), representing the relative growth volume of the R_e_ for every 10°C temperature increase as follows:

$$Q_{10}=\text{exp}(10b).$$ (2)

The daytime gaps in the CO_2_ flux (*F*_c_) during the growing season were filled through rectangular hyperbolic regression according to Formula (3) [25]:

$$F_{c}=\frac{F_{\text{max}}\;\alpha PPFD}{F_{\text{max}}+\;\alpha PPFD},$$ (3)

where PPFD is the photosynthetic photon flux density (μmol m^−2^ s^−1^), F_max_ is the NEE at a saturating light level (μmol CO_2_ m^−2^ s^−1^), and α is the apparent quantum yield (μmol CO_2_ μmol^−1^ photons).

### Vegetation measurement

The biomass and leaf area index (LAI) were measured six times over the whole growth season. The aboveground biomass measurement adopted the harvesting method, with five randomly collected samples, each including the vegetation within a square area covering 0.25 m^2^. The vegetation was cut and brought back to the lab with the roots to be dried in a 65°C thermostatic oven. The LAI was determined from measurements taken with a leaf area meter (LI−3100, Li−Cor). Based on plant phenology data from the TRSR, we assumed that the biomass and the LAI for the sampling field were both zero before April 20 (day of year (DOY) 111) and after October 18 (DOY 292), which marked the beginning and end of the growing season, respectively. LAI gaps were linearly interpolated to daily intervals [27].

### Path analysis

The relationships between the CO_2_ flux and environmental factors were evaluated using path analysis. Path analysis is the continuation of the simple correlation coefficient and decomposes the correlation coefficient based on multiple regression. It uses direct and indirect paths to indicate the direct effect of a variable on a dependent variable and the indirect effects of other variables on a dependent variable [28, 29]. Path analysis was performed using the standardization of multiple linear regression models that are included with the RGE and CORR packages of SAS 9.4 software (SAS Institute Inc., Cary, NC, USA).

The decision coefficient R^2^_(j)_ is often used to quantify the integrated determination effect of environmental factors (x_j_) on the ecosystem CO_2_ flux (y)[30]:

$$\begin{array}{lll} R_{\left(j\right)}^{2} & = & R_{j}^{2}+\sum_{j\neq i}R_{ji}^{2}\\ R_{j}^{2} & = & b_{j}^{*2}\\ R_{ji}^{2} & = & 2b_{j}^{*}r_{ji}b_{i}^{*} \end{array}$$ (4)

R^2^(j) contains not only the direct determination effect R^2^_j_ of x_j_ only but also the indirect determination coefficient ($\sum_{j\neq i}R_{ji}^{2}$) related to x_j_. Additionally, b*^2^_j_ represents the path coefficient, and r_ji_ represents the correlation. The x corresponding to the maximum value of R^2^_(j)_ has the maximum synthesis effect on y and is called the main decision-making factor. In contrast, the x corresponding to the minimum value of R^2^_(j)_ is called the main confined factor.

## Results

### Meteorological and biological factors

**Fig 1:**
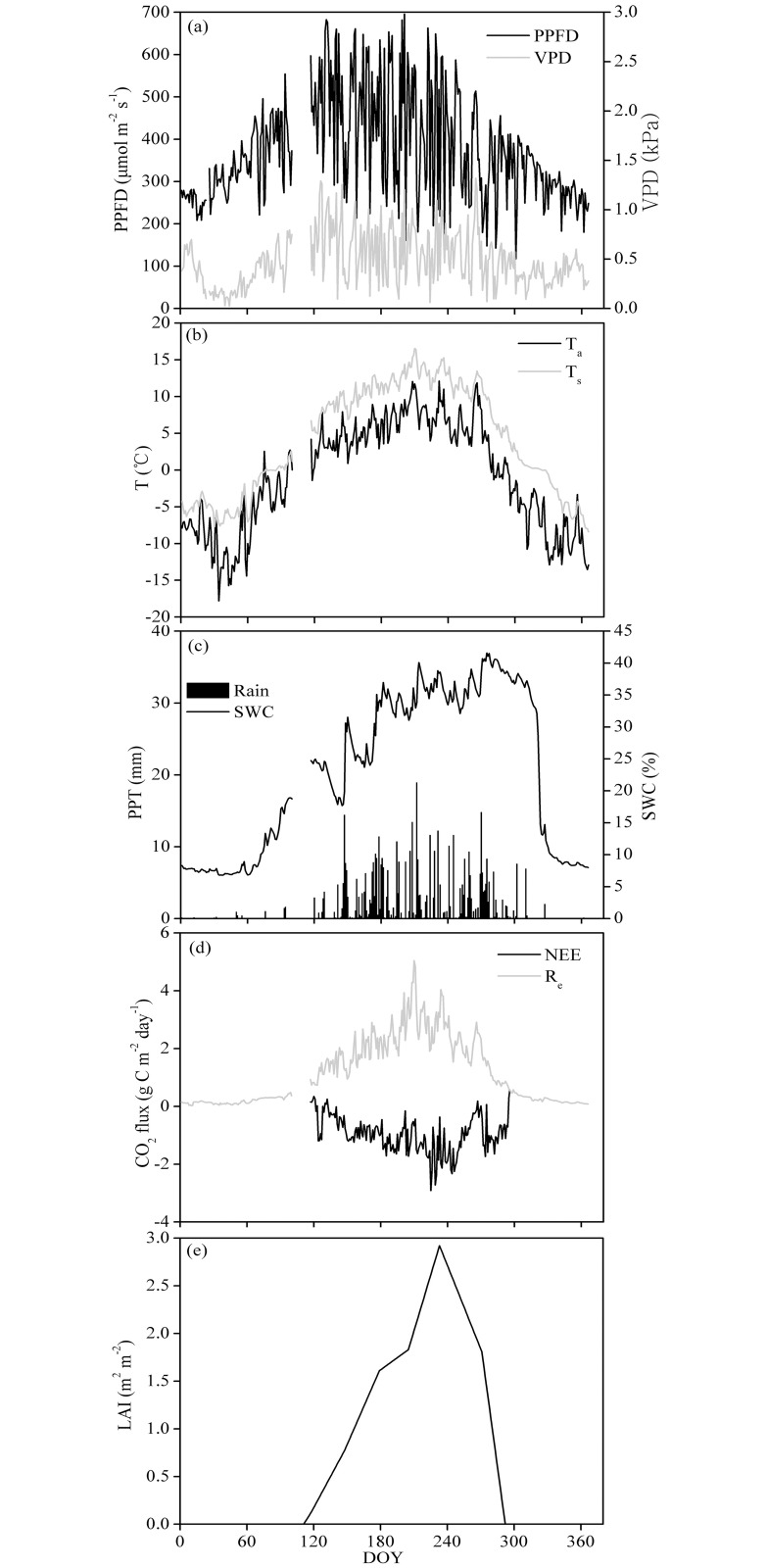
Temporal variation during 2008 in the (a) photosynthetic photon flux density (PPFD) and vapor pressure deficit (VPD), (b) daily mean air temperature (T_a_) and soil temperature at a depth of 5 cm (T_soil_), (c) daily precipitation (PPT) and soil water content (SWC) at a depth of 5 cm, (d) daily net ecosystem CO_2_ exchange (NEE) and ecosystem respiration (R_e_) and (e) leaf area index (LAI).

Fig 1a shows that the PPFD peaks occurred between May and August, when the solar elevation angle was higher than in other seasons. Because of the high elevation of the plateau, the PPFD values of the plateau also tended to be high, and the maximum daily values reached 695.9 μmol m^−2^ s^−1^ (Fig 1a). The vapor pressure deficit (VPD) also showed significant seasonal variation, reaching its highest and lowest values, approximately 1.32 and 0.04 kPa, respectively, during the growing season and during the winter (Fig 1a). The daily mean T_a_ and T_soil_ values showed the same seasonal variation trends, ranging from −17.8 to 12.1°C for T_a_ and from −8.4 to 16.5°C for T_soil_. The annual average was −0.54°C for T_a_ and 4.2°C for T_soil_ (Fig 1b). The annual PPT was 628.9 mm, which was higher than the average PPT across multiple years (approximately 500 mm), and the PPT during May–September accounted for 66.4% of the annual PPT. After October, the PPT was significantly reduced (Fig 1c). The variation in the soil water content (SWC) was strongly dependent on the PPT; the SWC was higher from May to October than at other times of the year and was generally maintained above 20% (Fig 1c).

Fig 1e shows that the LAI for the field in the sampled pasture started to increase at the end of April and reached the maximum LAI (2.9±0.3) at the end of August. In September, the LAI decreased rapidly because of leaf aging. The growing season for the pasture in 2008 (DOY 113–292) could be divided into the following four periods (Table 1): the beginning growing period (I, DOY 113–145), the fast growing period (II, DOY 146–194), the peak growing period (III, DOY 195–252) and the aged growing period (IV, DOY 253–292).

**Table 1:** Parameters describing the characteristics of the relationship between the daytime NEE and the incident PPFD (Formula (3)).

Treatment	DOY	LAI (m^2^ m^−2^)	SWC (%)	T_a_ (°C)	VPD (kPa)	α (μmol μmol^−1^)	F_max_ (μmol CO_2_ m^−2^ s^−1^)	R_e_ (μmol CO_2_ m^−2^ s^−1^)	n	R^2^	P value
Beginning growing period	113–145	0.35±0.06	22.88±3.59	4.02±1.86	0.52±0.29	−0.0022±0.0008	−5.22±0.34	0.21±0.04	16	0.93	<0.0001
Fast growing period	146–194	0.97±0.16	25.50±2.55	7.08±2.19	0.46±0.23	−0.0185±0.0053	−6.97±0.24	0.80±0.18	16	0.97	<0.0001
Peak growing period	195–252	1.92±0.25	35.01±2.47	9.59±2.95	0.46±0.12	−0.0358±0.0091	−8.69±0.66	1.51±0.23	16	0.98	<0.0001
Aged growing period	253–292	1.38±0.35	37.03±3.63	7.32±1.16	0.56±0.23	−0.0122±0.0047	−6.95±0.53	0.66±0.12	16	0.96	<0.0001
Entire growing season	113–292	1.51±0.45	31.72±2.73	7.38±2.41	0.49±0.24	−0.0275±0.0048	−7.86±0.73	1.79±0.28	16	0.98	<0.0001
SWC≤25%						−0.0092±0.0022	−4.28±0.66	0.94±0.18	16	0.96	<0.0001
25%<SWC≤30%						−0.0258±0.0046	−5.81±0.82	1.59±0.22	16	0.98	<0.0001
SWC>30%						−0.0329±0.0058	−9.51±0.31	2.08±0.18	16	0.99	<0.0001
T_a_≤5°C						−0.0220±0.0039	−5.89±0.41	1.25±0.16	16	0.97	<0.0001
5°C<T_a_≤15°C						−0.0289±0.0202	−8.02±1.80	1.69±0.26	16	0.97	<0.0001
T_a_>15°C						−0.0174±0.0054	−6.87±0.50	1.26±2.16	16	0.99	<0.0001
VPD≤0.6 kPa						−0.0291±0.0058	−8.85±1.26	1.85±0.11	16	0.99	<0.0001
VPD>0.6 kPa						−0.0112±0.0045	−8.20±0.43	1.23±0.38	16	0.97	<0.0001

### Diurnal course of CO_2_ exchange

**Fig 2:**
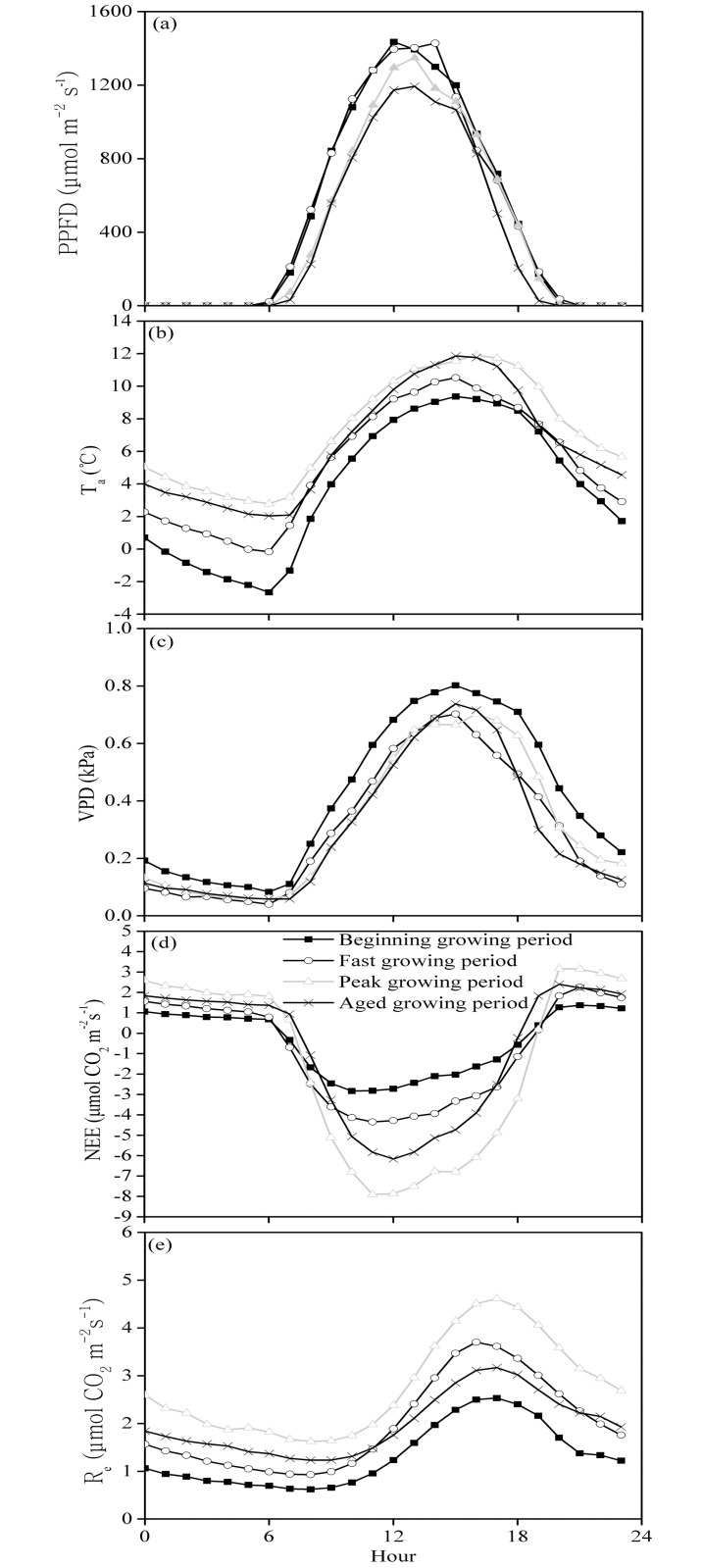
Average diurnal cycles of the (a) photosynthetic photon flux density (PPFD), (b) air temperature (T_a_), (c) vapor pressure deficit (VPD), (d) net ecosystem CO_2_ exchange (NEE) and (e) ecosystem respiration (R_e_) in different periods of the growing season. Bars indicate ± standard error (SE). The time zone is Beijing Standard Time (BST).

Fig 2d shows that the daily variation in the NEE was regular during every growth period. This variation was most likely a reflection of daytime absorption and nighttime emission. In the morning, the NEE was converted from a positive value (representing carbon emission) to a negative value (representing carbon absorption). The absorption value reached its maximum before noon (10:00–11:00 h) and then started to diminish. Near evening (approximately 19:00 h), the NEE changed from a negative value to a positive value. The hourly maximum and minimum NEE rates, which were 3.25 and −7.89 μmol CO_2_ m^−2^ s^−1^, respectively, both occurred during the peak growing period. Fig 2e shows that the hourly maximum R_e_ rate of the pasture, which occurred at approximately 16:00 h during the peak growing period, was 5.03 μmol CO_2_ m^−2^ s^−1^.

### Seasonal course of CO_2_ exchange

**Fig 3:**
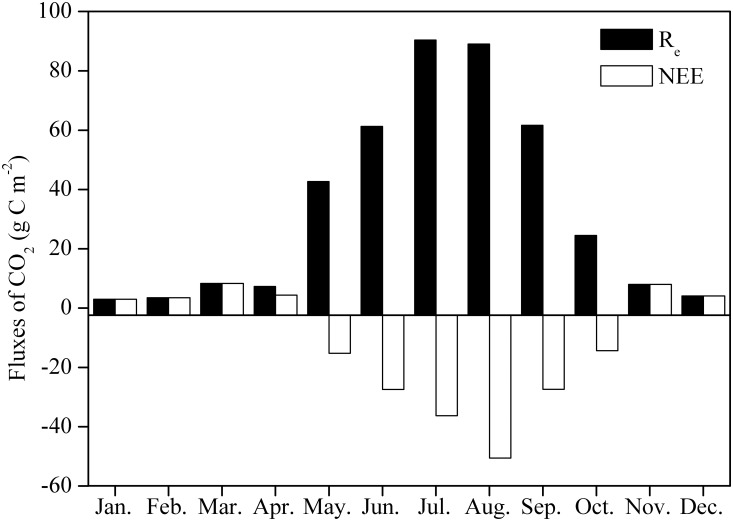
Seasonal variation in the monthly net ecosystem CO_2_ exchange (NEE) and in the ecosystem respiration (R_e_) in the studied cultivated pasture ecosystem in 2008.

Fig 1d shows that from January until the end of April, the pasture NEE was greater than 0 because the aboveground vegetation had withered; thus, the ecosystem was emitting carbon (NEE>0). Starting on May 1 (DOY 121), as the vegetation began to appear, the NEE began to drop to below 0. The whole ecosystem converted from carbon emission to carbon absorption (NEE<0) and reached peak carbon absorption between July and August. Starting in September, as the vegetation aged, the carbon absorption capability of the pasture gradually degraded. By the end of October, the NEE began to exceed 0, and the whole ecosystem engaged in carbon emission (NEE>0). The maximum daily absorption value, −2.91 g C m^−2^ day^−1^, occurred on August 12 (DOY 225). The ecosystem appeared to be a carbon sink during May−October (NEE<0), with the maximum CO_2_ uptake occurring in August, i.e., −50.59 g C m^−2^ month^−1^ (Fig 3). The annual NEE for the pasture in 2008 was −140.04 g C m^−2^ year^−1^. Thus, the pasture was a carbon sink during 2008.

There is a significant seasonal change in R_e_ for the cultivated pasture. Fig 3 shows an increasing trend in CO_2_ emissions from winter (January) to summer (July**−**August) and a decreasing trend until autumn (September), with maximum and minimum values in July (90.36 g C m^**−**2^ month^**−**1^) and January (2.96 g C m^**−**2^ month^**−**1^), respectively (Fig 3). In 2008, the daily maximum R_e_ for the pasture was 5.04 g C m^−2^ day^−1^ on July 28 (DOY 210) (Fig 1(e)). The annual R_e_ was 403.57 g C m^−2^ year^−1^ in 2008, of which approximately 85.5% fell in the growing season, from May to September.

### The relationship between the daytime NEE and the PPFD

**Fig 4:**
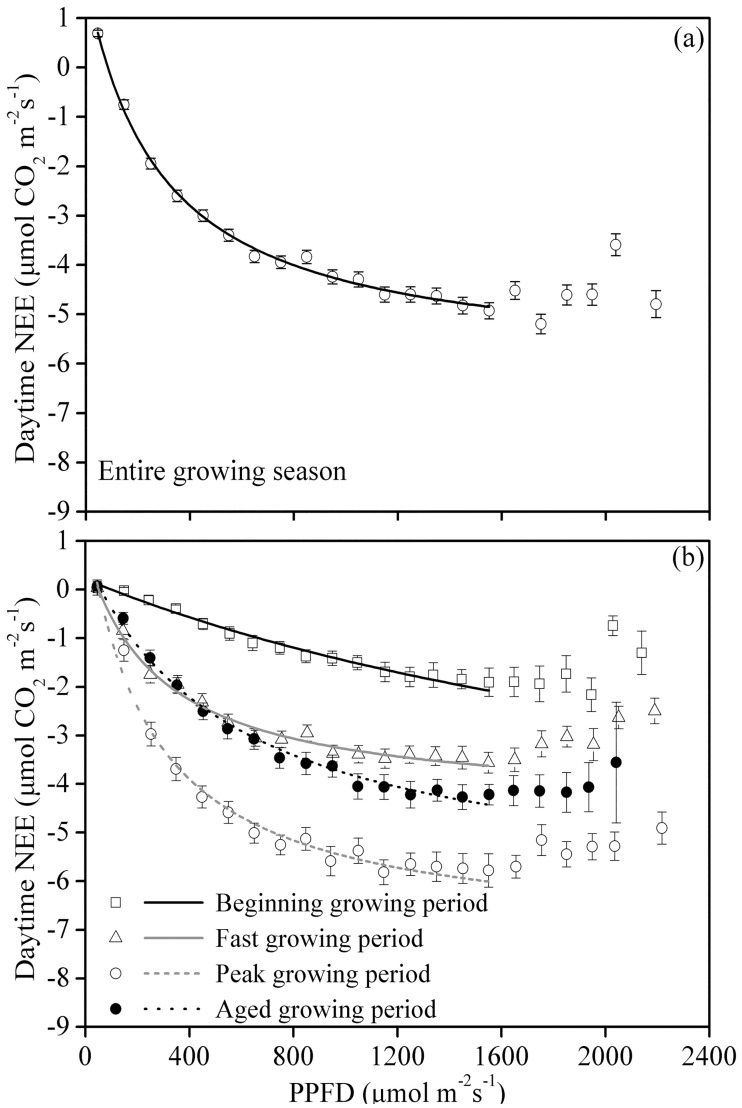
Relationship between the NEE and PPFD for (a) the entire growing season and (b) different periods of the growing season. The daytime net ecosystem CO_2_ exchange (NEE) data were averaged over PPFD bins of 100 μmol m^−2^ s^−1^. Bars indicate ±SE. Formula (3) was used to fit the data when the PPFD was below 1,600 μmol m^−2^ s^−1^; the regression coefficients are presented in Table 1.

We used Formula (3) to depict the relationship between the daytime NEE and the PPFD. The NEE data were averaged using PPFD bins of 100 μmol m^−2^ s^−1^. As shown in Fig 4, at PPFD<1600 μmol m^−2^ s^−1^, the daytime NEE decreased as the PPFD increased. However, for PPFD>1600 μmol m^−2^ s^−1^, the daytime NEE increased as the PPFD increased (Fig 4). Therefore, Formula (3) was only valid for depicting the relationship between the NEE and the PPFD for PPFD<1600 μmol^−1^ m^−2^ s^−1^. During the entire growing season, the model-derived α and F_max_ values in the pasture increased as the canopy developed, and their maximum values occurred during the peak growing period, reaching −0.0358 μmol CO_2_ μmol^−1^ photons and −8.69 μmol CO_2_ m^−2^ s^−1^, respectively (Table 1). During the entire growing season, the α and F_max_ values in the pasture were −0.0275 μmol CO_2_ μmol^−1^ photons and −7.86 μmol CO_2_ m^−2^ s^−1^, respectively.

To further study the influence of environmental factors on the NEE−PPFD curve, we inspected the NEE−PPFD curves generated under different T_a_ conditions (T_a_≤5°C, 5°C<T_a_≤15°C and T_a_>15°C), SWC conditions (SWC≤25%, 25%<SWC≤30%, and SWC>30%) and VPD conditions (VPD≤0.6 kPa and VPD>0.6 kPa). Under the aforementioned micrometeorological conditions, the NEE could be further subdivided based on the PPFD (using 100 μmol m^−2^ s^−1^ PPFD subdivisions), and the NEE was then averaged for each PPFD level. Statistically, this method can reduce or offset the errors that occurred during measurement [25].

**Fig 5:**
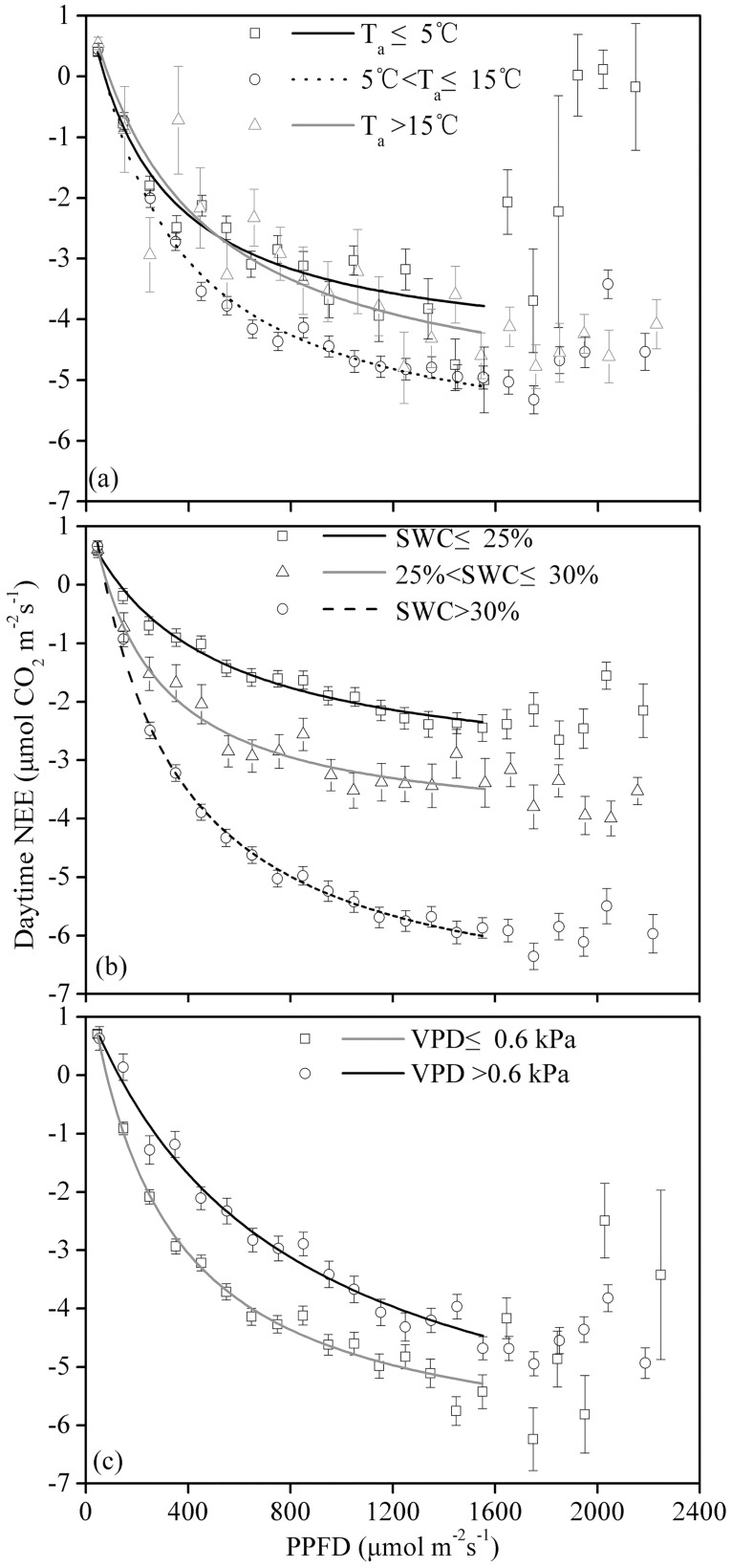
Relationship between the net ecosystem CO_2_ exchange (NEE) and PPFD for different values for (a) air temperature (T_a_), (b) soil water content (SWC) and (c) vapor pressure deficit (VPD). The daytime NEE data were averaged over PPFD bins of 100 μmol m^−2^ s^−1^. Bars indicate ±SE. Formula (3) was used to fit the data when the PPFD was below 1,600 μmol m^−2^ s^−1^; the regression coefficients are presented in Table 1.

In the pasture, the F_max_ and α values for the NEE−PPFD curve were under the influence of the SWC, T_a_ and the VPD. Both F_max_ and α increased as the SWC increased, and at a SWC<20%, F_max_ and α were significantly lower than they were when the SWC>30%. F_max_ and α were highest when 5°C<T_a_≤15°C. F_max_ and α decreased as the VPD increased, and at VPD>0.6 kPa, F_max_ and α were 93% and 38%, respectively, of their values at VPD≤0.6 kPa (Fig 5 and Table 1).

### The relationships between the daytime NEE and the values for T_a_, VPD and SWC

**Fig 6:**
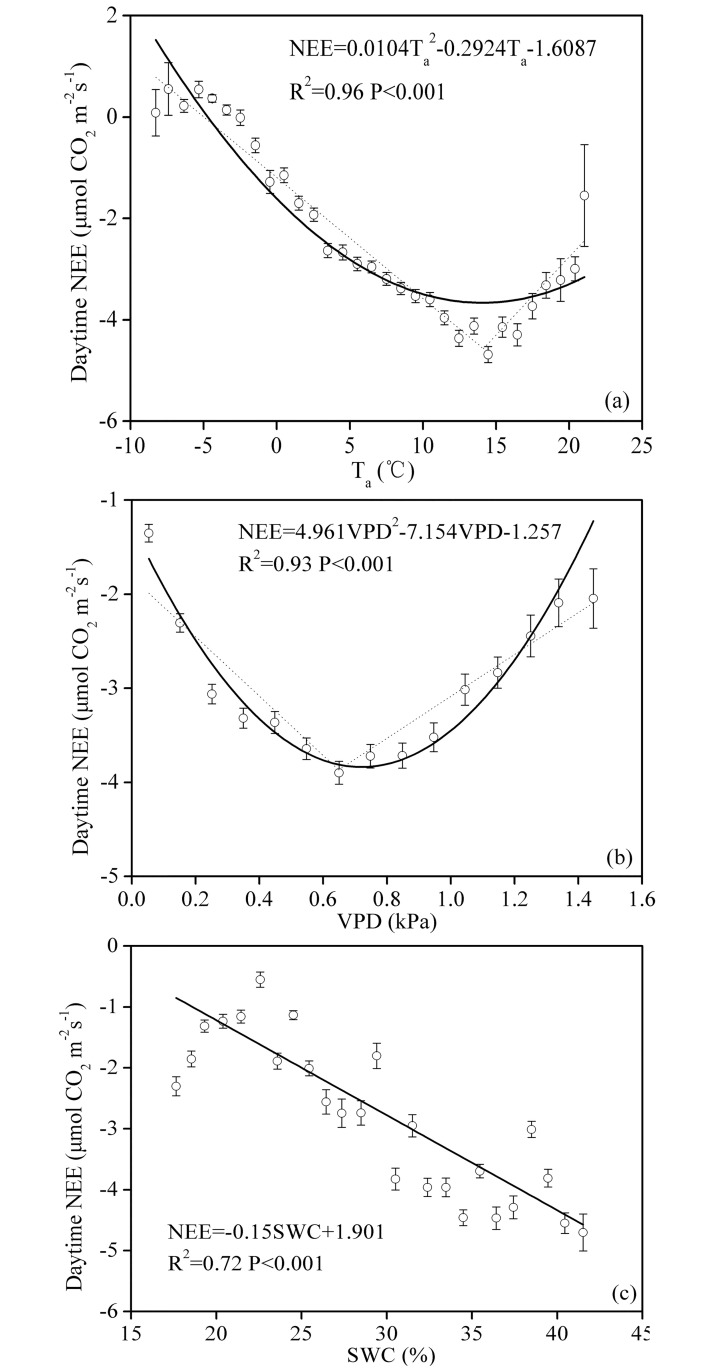
Relationships between the daytime NEE and (a) air temperature (T_a_), (b) vapor pressure deficit (VPD) and (c) soil water content (SWC) at a depth of 5 cm. The daytime NEE data were averaged over bin widths of 1°C for T_a_, 0.1 kPa for VPD and 1% for SWC. Bars indicate ±SE. The dotted lines in (a) and (b) were fitted using a piecewise regression model.

For statistical purposes, the daytime NEE data were averaged based on abiotic controls divided into bins, with bin widths of 1°C for T_a_, 1% for SWC and 0.1 kPa for VPD over all the PPFD values. Fig 6a and 6b show that the relationships between the daytime NEE and T_a_ and the VPD can be depicted by a quadratic function, and the stepwise regression analysis results indicated that the optimal T_a_ and VPD values for the pasture ecosystem were 14.1°C and 0.65 kPa. The daytime NEE decreased as the SWC increased (Fig 6c).

### R_e_ in response to T_soil_ and the SWC

**Fig 7:**
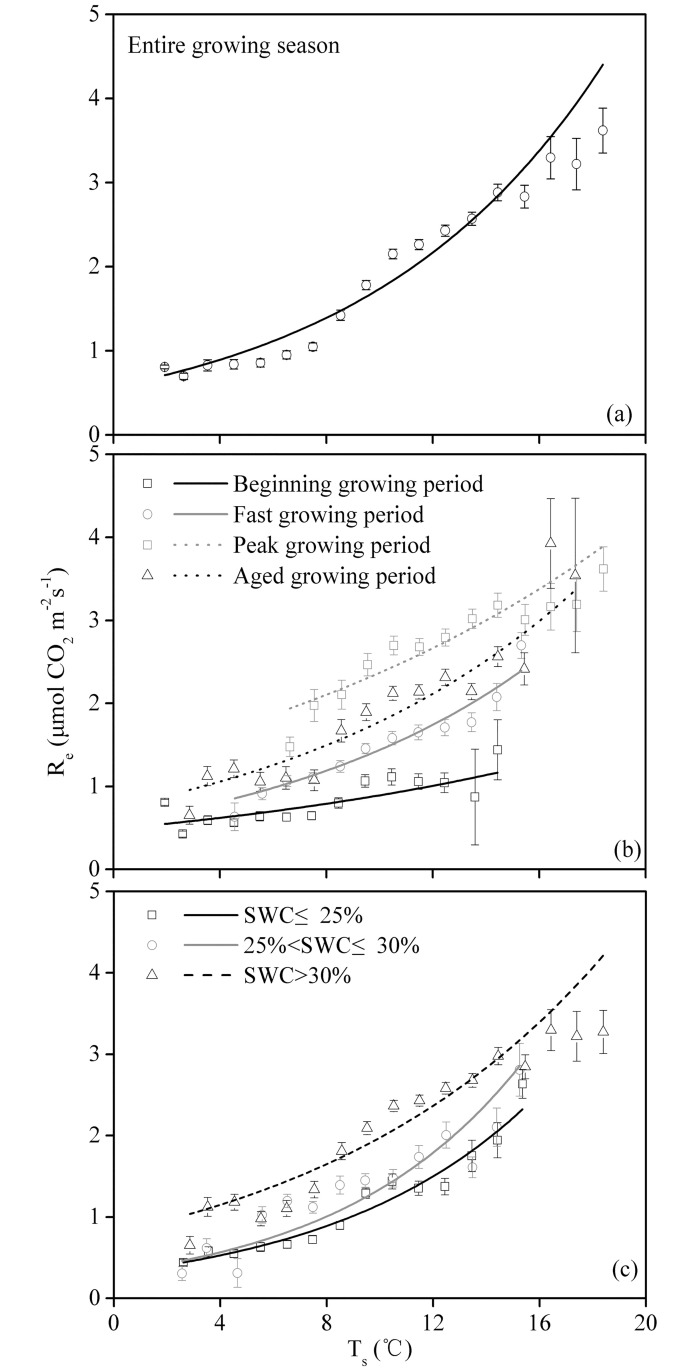
Relationship between the nighttime net ecosystem CO_2_ exchange (NEE) and soil temperature (T_soil_) for (a) the entire growing season, (b) the different growing periods and (c) different soil water content (SWC) ranges. The nighttime NEE data were averaged over a bin width of 1°C for T_soil_. Bars indicate ±SE. Formula (1) was used to fit the data; the regression coefficients are presented in Table 2.

For the entire growing season, the nighttime NEE data were bin averaged using T_soil_ bins of 1°C. Fig 6 shows that R_e_ increased exponentially as the temperature increased, and the Q_10_ for the pasture during the entire growth season was 3.0 (Fig 7 and Table 2), with values of 1.9, 2.9, 1.8 and 2.7 for the beginning, fast, peak and aged growing periods, respectively (Fig 7 and Table 2).

**Table 2:** Parameters describing the characteristics of the relationship between the nighttime NEE and T_soil_ (Formulas (1) and (2)).

Treatment	DOY	SWC	a	b	R^2^	Q_10_	P value
Beginning growing period	113–145	23.17±2.13	0.4661	0.0664	0.66	1.9425	<0.0001
Fast growing period	146–194	26.08±1.74	0.4809	0.1068	0.94	2.9096	<0.0001
Peak growing period	195–252	35.62±2.23	1.2801	0.0583	0.83	1.7914	<0.0001
Aged growing period	253–292	37.63±3.02	0.6434	0.0992	0.89	2.6966	<0.0001
Entire growing season	113–292	32.41±2.25	0.5551	0.1088	0.95	2.9683	<0.0001
SWC≤25%			0.3136	0.1304	0.96	3.6840	<0.0001
25%<SWC≤30%			0.3188	0.1438	0.79	4.2123	<0.0001
SWC>30%			0.6936	0.0960	0.91	2.6117	<0.0001

To further investigate the effect of the SWC on T_soil_ and R_e_, we investigated the T_soil_−R_e_ relation under different SWC conditions (SWC≤25%, 25%<SWC≤30% and SWC>30%). The results showed that the Q_10_ of the cultivated grassland reached its maximum at 25%<SWC≤30%.

### The relationship between the daily NEE and the LAI

**Fig 8:**
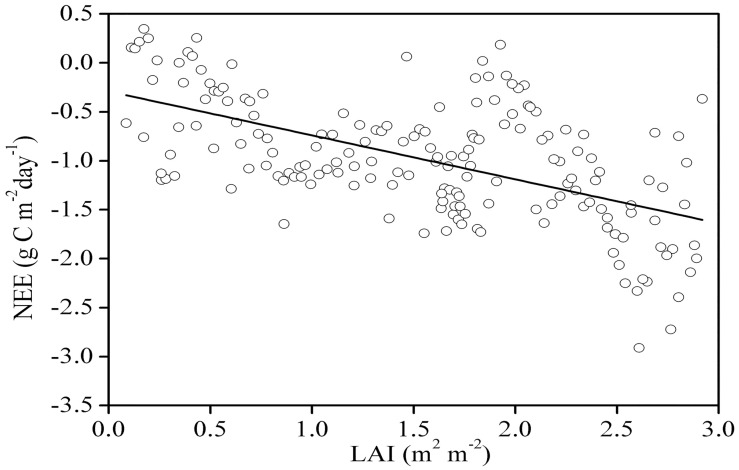
Relationship between the daily net ecosystem CO_2_ exchange (NEE) and the leaf area index (LAI) from April 20 to October 18, 2008.

Fig 8 indicates that during the entire growing season, the cultivated grassland’s daily integrated NEE and LAI showed a linear relationship in which NEE = (−0.449± 0.005)×LAI−(0.291±0.008), n = 175, adjusted R^2^ = 0.325 and F = 84.8. Therefore, 32.5% of the variation in the NEE could be explained by variation in the LAI.

### Path analysis evaluation of the daily NEE

**Fig 9:**
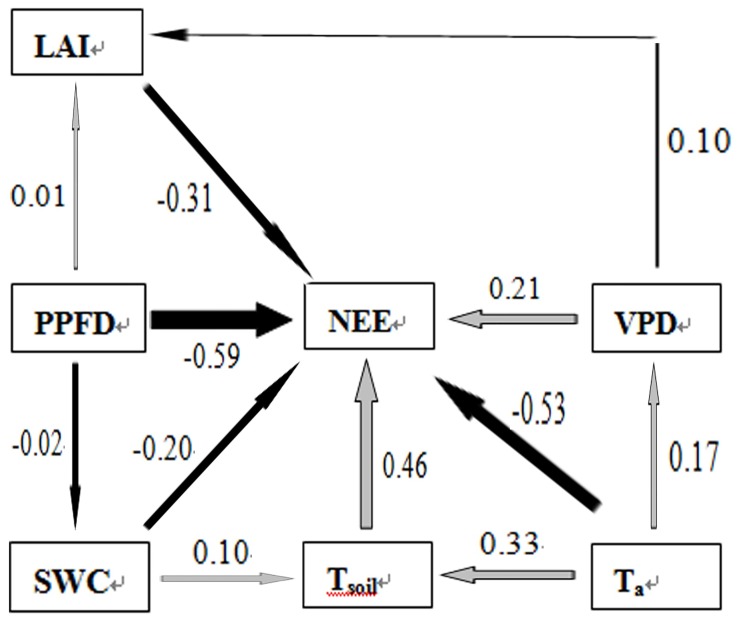
Path diagrams showing the effects of air temperature (T_a_), soil temperature at a depth of 5 cm (T_soil_), soil water content (SWC) at a depth of 5 cm, leaf area index (LAI), the photosynthetic photon flux density (PPFD) and the vapor pressure deficit (VPD) on daily NEE during the growing season in the studied pasture. The thickness of each arrow and the value beside it indicate the path coefficient. Black arrows show negative correlations, and gray arrows indicate positive correlations. The analysis was based on the daily average values of all variables.

The path analysis results show that during the entire growing season, the relationship between the cultivated grassland’s daily integrated NEE and environmental factors can be described using the following formula: NEE = 2.22778−0.01531×LAI−0.00331×PPFD+0.46160×T_soil_−0.34505×T_a_−10.45735×SWC+2.11380×VPD, with n = 1071, adjusted R^2^ = 0.6025 and F<0.0001. The direct path coefficients (R^2^_j_) of the environmental factors (x_j_) affecting the daily integrated NEE (y) are ranked as follows: T_soil_ (0.46)>VPD (0.21)>SWC (−0.20)>LAI (−0.31)>T_a_ (−0.53)>PPFD (−0.59) and for the decision coefficient R^2^(j), T_soil_ (0.12)>SWC (−0.18)>LAI (−0.20)>VPD (−0.34)>T_a_ (−0.44)>PPFD (−0.69) (Fig 9).

## Discussion

### The effect of biotic and abiotic controls on the NEE

The maximum F_max_ for the pasture ecosystem (−8.69 μmol CO_2_ m^−2^ s^−1^) occurred during the peak growing period (Table 1) and was nearly identical to the F_max_ for a steppe−*Kobresia* meadow during the peak growing period (−8.7 μmol CO_2_ m^−2^ s^−1^) [11]. However, the pasture maximum F_max_ was lower than the values reported for other grassland ecosystems (from −9.6 to −40.2 μmol CO_2_ m^−2^ s^−1^) [27]. For the entire growing season, the α value for the pasture was 0.02754 μmol CO_2_ μmol^−1^ photons, which was higher than that for the steppe−*Kobresia* meadow (−0.0159 μmol CO_2_ μmol^−1^ photons) [11]. However, the α for the pasture for the entire growing season was at a moderate to low level compared with the values for other grassland and cropland ecosystems, as reported by Li et al. (2005) [27] (from −0.008 to −0.465 μmol CO_2_ μmol^−1^ photons). These findings indicate that the light-use efficiency of the pasture was low. This low efficiency was related to the use of C3 vegetation to establish the pasture and to the high elevation and low temperatures of the pasture ecosystem [31].

Under low T_a_ values (T_a_≤5°C), the F_max_ and α of the NEE−PPFD curve for the pasture were relatively low (Table 1), mainly because low temperature can suppress the activity of photosynthesis-related enzymes [32]. This situation was also observed in the desert steppe of Inner Mongolia [33]. At SWC<20%, the F_max_ and α values of the NEE-PPFD curve for the pasture were relatively low (Table 1), primarily because the low SWC can constrain plant growth. A similar situation also occurred for the steppe [27]. During the aged growing period, the F_max_ and α values of the NEE-PPFD curve for the pasture decreased significantly. This finding is related to the reduced chlorophyll content of older plants, which also show decreased activity of photosynthesis-related enzymes [34]. Similar results have also been reported for a fenced steppe [30].

The optimal T_a_ for CO_2_ uptake in the pasture was 14.1°C (Fig 5a), which was quite similar to that of an alpine meadow (15°C) [35]. In our study, T_a_ had marked effects on the NEE. The NEE decrease at lower temperatures was most likely caused by the slow growth rate during the early and late stages of the growing season, whereas the depression of the NEE at relatively higher temperatures could be ascribed primarily to enhanced respiration and depressed plant photosynthesis in response to high temperatures and high radiation levels [36].

In many ecosystems, moisture is an important factor that influences the daytime NEE. The daytime NEE for the pasture decreased with increases in the soil moisture (Fig 5c). This trend indicated that increased soil moisture can improve the carbon absorption capability of the pasture. Similar results have been reported for a Mongolian steppe [27]. Preliminary studies demonstrated that a lack of moisture could result in the closure of plant stomata, further reducing plant CO_2_ absorption. In addition, stomatal closure had a significant effect on leaves. An increasing leaf temperature can enhance leaf photorespiration, which further reduces CO_2_ acquisition by the plants.

In the present study, the daytime NEE and the VPD of the pasture were quadratically related (Fig 5b), and similar results were observed for a temperate desert steppe [33]. The daytime suppression of the NEE by a high VPD could be primarily attributed to the physical relationship between the temperature and the VPD (Fig 2). Because this relationship can affect the hydraulic status of plants and leaves, leading to leaf closure, it can affect the acquisition of CO_2_ by plants [37].

The process of carbon exchange between plants and the atmosphere is jointly regulated by multiple environmental factors (such as the PPFD, T_a_, SWC and VPD); thus, it is difficult to identify a specific effect on the NEE caused by a single factor, especially between T_a_ and the VPD, given that a rising T_a_ is always associated with an increased VPD. Therefore, future studies of the response mechanisms of the NEE to environmental factors should use both modeling and multivariate analysis.

### The effect of T_soil_ and the SWC on the nighttime NEE

R_e_ is affected by multiple environmental and biological factors, and T_soil_ and the SWC can be regarded as controls [38].

R_e_ shows an exponential function with increasing temperature [39]. During the entire growing season in the present study, the Q_10_ for the pasture was 3.0 (Fig 7a and Table 2), which was higher than the Q_10_ value for low-elevation grassland ecosystems around the world (2.1 according to Zheng et al., 2009 [40]). A previous study showed that the Q_10_ value for R_e_ decreases as the temperature increases [40]. Here, the relatively high Q_10_ value for the TRSR could result from the low temperature on the plateau. Therefore, the results of the current study indicated that in the context of global warming, the TRSR pasture has a relatively strong carbon emission potential.

The Q_10_ value reached its maximum at a medium SWC (Fig 7c and Table 2). This situation also occurred in a *Stipa krylovii* steppe [41]. At a high SWC, the soil moisture can hinder the diffusion of O_2_. Therefore, a high SWC can suppress the decomposition of organic matter and decrease the microbial respiration rate. Under these conditions, the CO_2_ release and temperature are not sensitive, and the Q_10_ value is relatively low. However, at a low SWC, the primary component, composed of R_e_, derives from the more recalcitrant carbon material, and the Q_10_ of this material is low [42]. The situations discussed above cannot explain the high Q_10_ value during the aged growing period (SWC>30%). This result indicated that plant phenology was another factor affecting the Q_10_. A similar result has also been reported for a desert steppe [33].

### The effect of the LAI on the daily NEE

The structure of the plant canopy, especially the leaf area and light interception capability, determine the quantity of radiation absorbed and reflected by the plant canopy. Therefore, these factors can have a direct influence on plant photosynthesis [43, 44]. For the pasture ecosystem, the LAI could explain 32.5% of the NEE variation (Fig 8), and this percentage of explained variation was higher than that found for a desert steppe (26%, [33]). This finding could be attributed to the additional PPT received by the pasture ecosystem during the growing season.

### Effects of environmental factors on the CO_2_ flux

In the present study, path analysis was conducted to evaluate the relationships between various environmental factors and the NEE in the studied pasture during the growing season (Fig 9). Among the six factors directly affecting the NEE, the path coefficient for T_soil_ in the pasture was 0.46, which was much higher than the contributions of the other factors. T_soil_ explained most of the variability in the daily average NEE in the pasture. This conclusion agrees with the conclusions of Wang et al. (2011) [30]. Temperature is an important factor regulating several ecological processes and properties associated with the CO_2_ flux and its response to climate variation, including the evapotranspiration rate [45], canopy development [46] and SWC [47]. However, the minimum value of the decision coefficient R^2^_(j)_ (the synthesis index), which indicates the importance of the PPFD on the NEE, was −0.69 in the pasture. Therefore, the PPFD was the main factor, although it must be limited to lower values for dynamic ecosystem CO_2_ uptake. Similar results have been found for subalpine environments [28] because light is the most important ecological factor for regulating plant photosynthesis; both high and low light intensity can limit plant absorption and fix CO_2_ [48]. On the one hand, due to the high altitude in the TRSR, the PPFD was often greater than 1600 μmol m^−2^ s^−1^ at around noon on clear days, exhibiting a well-defined inhibitory effect on plant photosynthesis (Fig 4). On the other hand, the growing season on the Qinghai−Tibet Plateau coincides with precipitation and heat, and 69% of the days during the growing season of 2008 were rainfall days (Fig 1c). Constant rainfall reduces the time and intensity of sunlight. According to the statistics, the ecosystem daily cumulative PPFD value was less than 30 mol m^−2^ day^−1^, accounting for 31% of the total growing season days (Fig 1c). According to Satio et al., (2009) [29], low PPFD values (daily PPFD cumulative values less than 30 mol m^−2^ day^−1^) are important photosynthesis inhibition factors for Qinghai−Tibetan grassland ecosystems. Moreover, Satio et al. [29] also found that the most conducive daily PPFD cumulative value for plant photosynthesis is approximately 50 mol m^−2^ day^−1^ on the Qinghai−Tibetan Plateau (Fig 1(b)), although this phenomenon only occurs over 5% of the growing season (Fig 1b). Therefore, the special geographical environment (high altitude) and climatic conditions (precipitation and heat simultaneously) are the main reasons for the PPFD, becoming the main factor constricting NEE in the ecosystem.

### Diurnal and seasonal variation in the NEE and R_e_

At various stages during the growing season, the carbon absorption of the pasture ecosystem was significantly stronger at noon than before noon, indicating that the daily NEE is significantly suppressed around noon (Fig 2d). Fu et al. (2006) [36] also obtained a similar asymmetrical distribution of the NEE in a study of an alpine shrub. Because of photosynthetic depression at high temperatures, as well as stomatal closure at high PPFD levels, the carbon assimilation was severely restricted at noon and during the early afternoon. For most plants on the Qinghai−Tibetan Plateau, the photosynthetic depression at noon is a common phenomenon. This response of the plants is primarily due to enhanced respiration and depressed photosynthesis at high temperatures under high-radiation conditions [36].

For the pasture ecosystem, the magnitude of the maximum hourly NEE was −7.89 μmol CO_2_ m^−2^ s^−1^, and this value was lower than that of other grassland ecosystems located at similar latitudes, such as the tall prairie grassland native to North America (−23 μmol CO_2_ m^−2^ s^−1^) [49], other prairie plains in the USA (−19.5 μmol CO_2_ m^−2^ s^−1^) [50], and the alpine meadow at Haibei Station (−10.8 μmol CO_2_ m^−2^ s^−1^) [51]. This lower magnitude of the NEE in the current study could be attributed to the relatively low temperature of the pasture ecosystem and to the C3 composition of most of the pasture vegetation.

For the pasture ecosystem, the magnitude of the maximum daily NEE was −2.91 g C m^−2^ day^−1^, which is at the lower end of the maximum daily NEE variation range (from −1.91 to −9.3 g C m^−2^ day^−1^) for other grassland ecosystems [27]. This relatively low value was related to the practice of single-species sowing, which greatly reduces the plant diversity [52]. Naeem et al. (1994) [53] found that a reduction in plant diversity can cause a simplification in the canopy structures and a reduction in the light acquisition and utilization efficiency of a plant colony, thereby reducing the CO_2_ uptake of the overall ecosystem.

The annual NEE of the pasture in 2008 indicated that the pasture acted as a medium-strength carbon sink compared with other grassland ecosystems (from −18 to −274 g C m^−2^ year^−1^) [27]. The low T_a_ in the environment and the matching traits of the cool-adapted plants (e.g., the depression of the NEE at relatively higher temperatures) might have operated as important environmental restrictions on the potential of the pasture to act as a carbon sink.

The plant pasture showed a significant seasonal change in CO_2_ emissions, with the maximum value occurring during the peak growing period (Fig 3). Similar patterns have been found for a native alpine meadow [16]. Li et al. (2015) [16] attributed this change to high temperatures and biomass during the peak growing period. For the pasture ecosystem, the annual R_e_ in 2008 was 403.57 g C m^−2^ year^−1^, which was lower than that in a native alpine meadow near Haibei Station (488.5−555.6 g C m^−2^ year^−1^) [16, 54] and was similar to or less than other values (from 138 to 2392 g C m^−2^ year^−1^) for other grassland ecosystems [55]. This finding indicates that the construction of cultivated grassland will not increase CO_2_ emissions in the TRSR.

## Conclusion

We adopted eddy covariance to investigate the NEE for a single-sown cultivated pasture of *Elymus nutans* in the TRSR in 2008. Our results show that for the pasture, the NEE for the entire year was 140.01 g C m^−2^ year^−1^. Therefore, the cultivated grassland was a carbon sink during 2008. Because of the low temperatures in the TRSR, the annual R_e_ of the pasture was only 403.57 g C m^−2^ year^−1^ in 2008, lower than those in most grassland ecosystems around the world. This finding implies that cultivated grassland establishment can both effectively resolve the grass−livestock conflict and properly improve the grassland carbon fixation capacity in the TRSR. Moreover, the Q_10_ value in the pasture for the entire growing season was 3.0, which was higher than that in low-elevation grassland ecosystems around the world, indicating greater sensitivity to elevated temperatures in the future in terms of ecosystem carbon loss in the study area. During the daytime, the NEE was primarily regulated by the PPFD; at night, the NEE was mainly regulated by T_soil_. A higher temperature can suppress photosynthesis in pastures, reducing the carbon absorption capacity of pasture ecosystems. The daily NEE and LAI were linearly related, and 32.5% of the NEE variation can be interpreted based on the LAI variation. Path analysis showed that the daily NEE in the growing season of cultivated grassland was controlled by various ecological factors at the same time. Among them, T_soil_ was found to be the main decision-making factor for the daily NEE, and PPFD is the main constraining factor of the NEE in the studied ecological system.

## Supporting Information

S1 File Language polish editing certificate. (PDF)

S1 Table Temporal variation in meteorological and biological factors and CO_2_ exchange during 2008. Abbreviations: PPFD, photosynthetic photon flux density; VPD, vapor pressure deficit; T_a_, daily mean air temperature; T_soil_, soil temperature at a depth of 5 cm; PPT, daily precipitation; SWC, soil water content at a depth of 5 cm, NEE, daily net ecosystem CO_2_ exchange; R_e_, ecosystem respiration; and LAI, leaf area index (LAI).
(XLSX)

S2 Table Diurnal variation in meteorological and CO_2_ exchange (PPFD, T_a_, VPD, R_e_ and NEE). Abbreviations and symbols are as described for S1 Table.
(XLSX)

S3 Table The relationship between the daytime NEE and the PPFD. Abbreviations and symbols are as described for S1 Table.
(XLSX)

S4 Table Relationship between the NEE and environmental factors. Abbreviations and symbols are as described for S1 Table.
(XLSX)

S5 Table Relationship between R_e_ and T_soil_. Abbreviations and symbols are as described for S1 Table.
(XLSX)

S6 Table Path coefficients between the NEE and environmental factors in the studied pasture. Abbreviations and symbols are as described for S1 Table.
(XLSX)

## References

[1] Adams JM, Faure HFDL & Woodward FI. Increases in terrestrial carbon storage from the last glacial maximum to the present. Nature. 1990; 348: 711–714.

[2] Mcfadden JP, Eugster W, Chapin FS. A regional study of the controls on water vapor and CO_2_ exchange in arctic tundra. Ecology. 2003; 84: 2762–2776.

[3] Novick KA, Stoy PC, Katul GG, Ellsworth DS, Siqueira MBS, Juang J, et al Carbon dioxide and water vapor exchange in a warm temperate grassland. Oecologia. 2004; 138: 259–274. 10.1007/s00442-003-1388-z 14628214

[4] Wohlfahrt G, Anderson—Dunn M, Bahn M, Balzarolo M, Berninger F, Campbell C, et al Biotic, abiotic, and management controls on the net ecosystem CO_2_ exchange of European mountain grassland ecosystems. Ecosystems. 2008; 11: 1338–1351.

[5] Feiziene D, Feiza V, Kadziene G, Vaideliene A, Povilaitis V, Deveikyte I, et al CO_2_ fluxes and drivers as affected by soil type, tillage and fertilization. Acta Agr Scand B-S P. 2012; 62 (4): 311–328

[6] Fu Y, Zheng Z & Yu GR. Environmental influences on carbon dioxide fluxes over three grassland ecosystems in China. Biogeosciences. 2009; 6: 2879–2893.

[7] Hao Y B, Wang YF, Sun XM, Huang XZ, Cui XY, et al Seasonal variation in carbon exchange and its ecological analysis over *Leymus chinensis* steppe in Inner Mongolia. Sci China Ser D. 2006; 49: 186–195.

[8] Feng S, Tang MC & Wang DM. New evidence for the Qinghai—Tibetan Plateau as promoter region of climate change in our country. Chinese Sci Bull. 1998; 43: 633–636.

[9] Kang XC. Characteristics of climatic changes over last 40 years in Qinghai-Xizang plateau. J Glaciol Geocryol. 1996; 18: 281–288.

[10] Zhao L, Li YN, Xu SX, Zhou HK, Gu S, Yu GR, et al Diurnal, seasonal and annual variation in net ecosystem CO_2_ exchange of an alpine shrubland on Qinghai-Tibetan plateau. Global Change Biol. 2006; 12: 1940–1953

[11] Shi PL, Sun XM, Xu LL, Zhang XZ, He YT, Zhang DQ, et al Net ecosystem CO_2_ exchange and controlling factors in a steppe―*Kobresia* meadow on the Tibetan Plateau. Sci China Ser D, 2006; 49: 207–218.

[12] Chen G M. The status of the degraded pasture and its strategies of management in black beach of the headwater region of the three river. Sichuan Caoyuan, 2005; 10: 37–44.

[13] Dong SK, Li JP, Li XY, Wen L, Zhu L, Li YY, et al (2010) Application of design theory for restoring the “black beach” degraded rangeland at the headwater areas of the Qinghai-Tibetan Plateau. Afr J Agr Res. 2010; 5(25): 3542–3552.

[14] Gao XS, Tian ZC, Hao XN. The changes of alpine grassland soil nutrition at different deteriorate degree on high mountain meadow of three river source. J Qinghai Univ. 2006; 24: 37–40.

[15] Su XK, Wu Y, Dong SK, Wen L, Li YY, et al (2015) Effects of Grassland Degradation and Re-vegetation on Carbon and Nitrogen Storage in the Soils of the Headwater Area Nature Reserve on the Qinghai-Tibetan Plateau, China. Journal of Mountain Science. 2(3): 582–591.

[16] Li YY, Dong SK, Liu SL, Zhou HK, Gao QZ, Cao GM, et al Seasonal changes of CO_2_, CH_4_ and N_2_O fluxes in different types of alpine grassland in the Qinghai-Tibetan Plateau of China. Soil Biol Biochem. 2015; 80: 306–314.

[17] Xie ZB, Zhu JG, Liu G, Cadisch G, Hasegawa T, et al Soil organic carbon stocks in China changes from 1980s to 2000s. Global Change Biol. 2007, 13: 1989–2007.

[18] Wang QJ, Lai DZ, Jing ZC, Li SX, Shi HL. The present of resource, ecological environment and sustainable development in the source region of Yangtze, Yellow and Yalu Tsangpo rivers. J Lanzhou Univ. 2005; 41(4): 1–7.

[19] Dong SK Kang MY, Hu ZZ, Long R, Pu XP. Performance of cultivated perennial grass mixtures under different grazing intensities in the alpine region of the Qinghai-Tibetan Plateau. Grass Forage Sci. 2004; 59: 298–306.

[20] Dong SK, Long RJ, Hu ZZ, Kang MY (2005) Productivity and persistence of perennial grass mixtures under competition from annual weeds in the alpine region of the Qinghai-Tibetan Plateau. Weed Research 45: 114–120.

[21] Dong SK, Wen L, Li YY, Wang XX, Zhu L, Li XY. Soil Quality Effects of Grassland Degradation and Restoration on the Qinghai-Tibetan Plateau. Soil Sci Soc Am J. 2012; 76: 2256–2264.

[22] Webb E K, Pearman G I & Leuning R. Correction of flux measurements for density effects due to heat and water vapor transport. Q J Roy Meteor Soc. 1980; 106: 85–100.

[23] Wilson K, Goldstein A, Falge E, Aubinet M, Baldocchi D. Energy balance closure at FLUXNET sites. Agr Forest Meteorol. 2002; 113: 223–243.

[24] Gu S, Tang Y H, Du M Y, Kato T, Li YN, Cui XY, et al Short—term variation of CO_2_ flux in relation to environmental controls in an alpine meadow on the Qinghai—Tibetan Plateau. J Geophys Res. 2003; 108: 4670–4679.

[25] Falge E, Baldocchi D D, Olson R, Anthoni P, Aubinet M, Bernhofer C, et al Gap filling strategies for defensible annual sums of net ecosystem exchange. Agr Forest Meteorol. 2001; 107: 43–69.

[26] Lloyd J & Taylor JA. On the temperature dependence of soil respiration. Funct Ecol. 1994; 8, 315–323.

[27] Li SG, Asanuma J, Eugster W, Kotani A, Liu JJ. Urano T, et al Net ecosystem carbon dioxide exchange over grazed steppe in central Mongolia. Global Change Biol. 2005; 11: 1–15.

[28] Huxman TE, Turnipseed AA, Sparks JP, Sparks JP, Harley PC, Monson RK. Temperature as a control over ecosystem CO_2_ fluxes in a high-elevation, subalpine forest. Oecologia. 2003; 134: 537–546. 10.1007/s00442-002-1131-1 12647126

[29] Saito M, Kato T, Tang YH. Temperature controls ecosystem CO_2_ exchange of an alpine meadow on the northeastern Tibetan Plateau. Global Change Biol. 2009; 15, 221–228

[30] Wang YF, Cui XY, Hao YB, Mei XR, Yu GR, Huang XZ, et al The fluxes of CO_2_ from grazed and fenced temperate steppe during two drought years on the Inner Mongolia Plateau, China. Sci Total Environ. 2011; 410–411:182–190. 10.1016/j.scitotenv.2011.09.067 22024234

[31] Zhao L, Gu S, Zhou HK, Xu SX, Zhao XQ, Li YN. CO_2_ fluxes of artificial grassland in the Source Region of the Three Rivers on the Qinghai- Tibetan Plateau. Chinese J Plant Ecol. 2008; 32 (3): 544–554.

[32] Potvin C, Simon JP & Strain BR. Effect of low-temperature on the photosynthetic metabolism of the C-4 grass *Echinochloa-Crus-Galli*. Oecologia. 1986; 69: 499–506. 28311607 10.1007/BF00410354

[33] Yang FL, Zhou GS, Hunt JE, Zhang F. Biophysical regulation of net ecosystem carbon dioxide exchange over a temperate desert steppe in Inner Mongolia, China. Agr Ecosyst Environ. 2011; 142, 318–328.

[34] Kurahotta M, Satoh K & Katoh S. Relationship between photosynthesis and chlorophyll content during leaf senescence of rice seedlings. Plant Cell Physiol, 1987; 28: 1321–1329.

[35] Gu S, Tang Y, Du M, Cui XY, Kato T, Li YN, et al Effects of temperature on the CO_2_ exchange between the atmosphere and an alpine meadow. Phyton. 2005; 45: 361–370.

[36] Fu Y L, Yu G R, Sun X M, Li YN, Wen XF, Zhang LM, et al Depression of net ecosystem CO_2_ exchange in semi—arid *Leymus chinensis* steppe and alpine shrub. Agr Forest Meteorol. 2006; 137: 234–244.

[37] Souza RP, Machado EC, Silva JAB, Lagôaa AMMA, Silveirac JAG. Photosynthetic gas exchange, chlorophyll fluorescence and some associated metabolic changes in cowpea (*Vigna unguiculata*) during water stress and recovery. Environ Exp Bot. 2004; 51: 45–56.

[38] Hunt JE, Kelliher FM, McSeveny TM, Byers JN. Evaporation and carbon dioxide exchange between the atmosphere and a tussock grassland during a summer drought. Agr Forest Meteorol. 2002; 111: 65–82.

[39] Fang C & Moncrieff JB. The dependence of soil CO_2_ efflux on temperature. Soil Biol Biochem, 2001; 33:155–165.

[40] Zheng ZM, Yu GR, Fu YL, Wang YS, Sun XM, Wang YH. Temperature sensitivity of soil respiration is affected by prevailing climatic conditions and soil organic carbon content: a trans—China based case study. Soil Biol Biochem. 2009; 41: 1531–1540.

[41] Wang Y & Zhou G. Environmental effects on net ecosystem CO_2_ exchange at half-hour and month scales over *Stipa krylovii* steppe in northern China. Agr Forest Meteorol. 2008; 148: 714–722.

[42] Reichstein M, Tenhunen JD, Roupsard O, Ourcival JM, Rambal S, Miglietta F, et al Severe drought effects on ecosystem CO_2_ and H_2_O fluxes at three Mediterranean evergreen sites: revision of current hypotheses? Global Change Biol. 2002; 8, 999–1017.

[43] Ogren E. Convexity of the photosynthetic light-response curve in relation to intensity and direction of light during growth. Plant Physiol. 1993; 101: 1013–1019. 12231754 10.1104/pp.101.3.1013 PMC158720

[44] Baldocchi DD & Harley PC. Scaling carbon dioxide and water vapour exchange from leaf to canopy in a deciduous forest. II. Model testing and application. Plant Cell Environ. 1995; 18: 1157–1173.

[45] Polley HW, Mielnick PC, Dugas WA, Johnson HB, Sanabria J. Increasing CO_2_ from subambient to elevated concentrations increases grassland respiration per unit of net carbon fixation. Global Change Biol. 2006; 12:1390–1399.

[46] Wan SQ, Xia JY, Liu WX, Niu S. Photosynthetic overcompensation under nocturnal warming enhances grassland carbon sequestration. Ecology, 2009; 90: 2700–2710. 19886480 10.1890/08-2026.1

[47] Zhou X, Wan S, Luo Y. Source components and interannual variability in soil CO_2_ efflux under experimental warming and clipping in a grassland ecosystem. Global Change Biol. 2007; 13: 761–775.

[48] Schulze ED & Caldwell MM. Ecophysiology of Photosynthesis. Philadelphia: Springer-Verlag 1995.

[49] Ham JM & Knapp AK. Fluxes of CO_2_, water vapor, and energy from a prairie ecosystem during the seasonal transition from carbon sink to carbon source. Agr Forest Meteorol. 1998; 89, 1–14.

[50] Xu LK & Baldocchi DD. Seasonal variation in carbon dioxide exchange over a Mediterranean annual grassland in California, Agr Forest Meteorol. 2004; 123: 79–96.

[51] Kato T, Tang YH, Gu S, Cui XY, Hirota M, Du MY, et al Carbon dioxide exchange between the atmosphere and an alpine meadow ecosystem on the Qinghai—Tibetan Plateau, China. Agr Forest Meteorol. 2004; 124: 121–134.

[52] Wu GL, Liu ZH & Zhang L. Effects of cultivated grassland establishment on soil nutrients and carbon properties in a black—soil—type degraded grassland. Plant Soil. 2010; 333, 469–479.

[53] Naeem S, Thompson LJ, Lawler SP, Lawton JH, Woodfin RM. Declining biodiversity can alter the performance of ecosystems. Nature. 1994; 368: 734–736.

[54] Kato T, Tang Y H, Gu S, Hirota M, Du MY, Li NY, et al (2006) Temperature and biomass influences on interannual changes in CO_2_ exchange in an alpine meadow on the Qinghai-Tibetan Plateau. Global Change Biol. 2006; 12: 1285–1298.

[55] Kato T & Tang YH. Spatial variability and major controlling factors of CO2 sink strength in Asian terrestrial ecosystems: evidence from eddy covariance data. Global Change Biol. 2008, 14: 2333–2348.

